# How Lifestyle Changes during the COVID-19 Global Pandemic Affected the Pattern and Symptoms of the Menstrual Cycle

**DOI:** 10.3390/ijerph192013622

**Published:** 2022-10-20

**Authors:** Georgie Bruinvels, Richard C. Blagrove, Esther Goldsmith, Laurence Shaw, Daniel Martin, Jessica Piasecki

**Affiliations:** 1 Orreco, Ltd., London TW1 3DY, UK; 2 Faculty of Medical Sciences, University College London, London WC1E 6BT, UK; 3 School of Sport, Exercise and Health Sciences, Loughborough University, Loughborough LE11 3TU, UK; 4 School of Science and Technology, Nottingham Trent University, Nottingham NG11 8NS, UK; 5 School of Sport and Exercise Sciences, University of Lincoln, Lincoln LN6 7GA, UK

**Keywords:** female physiology, reproductive function, symptoms, eumenorrheic, nutrition, training

## Abstract

This research investigated the implications that the COVID-19 pandemic had on the menstrual cycle and any contributing factors to these changes. A questionnaire was completed by 559 eumenorrheic participants, capturing detail on menstrual cycle symptoms and characteristics prior to and during the COVID-19 pandemic lockdown period. Over half of all participants reported to have experienced lack of motivation (61.5%), focus (54.7%) and concentration (57.8%). 52.8% of participants reported an increase in cycle length. Specifically, there was an increase in the median cycle length reported of 5 days (minimum 2 days, maximum 32 days), with a median decrease of 3 days (minimum 2 days and maximum 17 days). A lack of focus was significantly associated with a change in menstrual cycle length (*p* = 0.038) reported to have increased by 61% of participants. Changes to eating patterns of white meat (increase *p* = 0.035, decrease *p* = 0.003) and processed meat (increase *p* = 0.002 and decrease *p* = 0.001) were significantly associated with a change in menstrual cycle length. It is important that females and practitioners become aware of implications of environmental stressors and the possible long-term effects on fertility. Future research should continue to investigate any long-lasting changes in symptoms, as well as providing education and support for females undergoing any life stressors that may implicate their menstrual cycle and/or symptoms.

## 1. Introduction

The menstrual cycle is a natural process for most women that occurs in the reproductive years between puberty and menopause. A ‘normal’ (or eumenorrheic) menstrual cycle lasts 22–35 days [1] and is characterised by cyclical fluctuations of sex hormones, mediated by the hypothalamic-pituitary axis, which is essential for maintenance of bone health and fertility. Whilst oestrogen and progesterone are the key sex hormones involved in the reproductive system, they are also vital in the regulation of other physiological systems and maintaining holistic health. In particular, oestrogen is a key regulator of bone resorption [2] and also has a cardioprotective role [3]. Therefore, the cyclical oestrogen exposure provided through the menstrual cycle, from puberty to pre-menopause, could also reduce the risk of other health conditions such as osteoporosis and cardiovascular disease [4,5].

A eumenorrheic menstrual cycle can be used to indicate that the body is in an adaptive physiological state, and not subject to excessive stress, defined as those who have had ≥9 menses and <17 within 12 months. Therefore, from a physical performance perspective, it can be used to guide readiness and adaptation, as well as determining a state of homeostasis. Dysregulation of this process may be multifactorial; often occurring as a result of perturbation of the hypothalamic-pituitary axis [6], which causes a reduction in the secretion and production of ovarian steroids [7]. Irregularities may sometimes present with anovulation and could also involve abnormal uterine bleeding and changes in cycle length or the cessation of cycles altogether (hypothalamic amenorrhea) [4,8]. Whilst prolonged energy deficiencies are often cited as being the aetiology of functional hypothalamic amenorrhea [9], other causes have been reported, such as sleep disturbances, lifestyle stress and alterations in circadian timing systems [9]. Up to 90% of women of a reproductive age also experience menstrual cycle symptoms [10], and severe symptoms have consistently been shown to affect quality of life [11,12] as well as negatively impacting exercise performance [13,14]. Premenstrual symptoms are positively associated with poor diet and lack of exercise [15,16], as well as increased alcohol intake [17].

Psychological stress has frequently been associated with alterations to menstrual cycle duration and the number and severity of symptoms [18,19]. One illustration of this is the observed increase in the prevalence of amenorrhea during wartime [20,21], although these periods in history were also associated with significant rationing on nutritional sources, another known factor linked to menstrual irregularities. Recent research has reported that students under a high amount of perceived stress were four times more likely to be amenorrhoeic [22]. Furthermore, women with stressful jobs were twice as likely to experience a decreased cycle length [23], often as a consequence of decreased follicular phase length, with little variation in the length of the luteal phase [24]. However, stressful environments have not consistently been shown to cause changes in the length of menstrual cycle characteristics [25,26,27]. Contradictions between findings may be due to individual variation in dispositional resilience, which may be a protective psychological trait regarding menstrual function in high stress scenarios [19]. High-quality data collection is challenging too, especially if ovulation monitoring is needed alongside menstrual cycle and symptom reporting.

Coronavirus disease 2019 (COVID-19) is a highly contagious and, for some, a fatal disease. The spread of COVID-19 at the beginning of 2020 resulted in a global pandemic and public health emergency. The pandemic had a widespread, negative impact on many aspects of society, with continuous uncertainty regarding health as well as changes to working and lifestyle patterns. The measures that were required to slow the spread of disease at the beginning of the pandemic, such as self-isolation and limits on exercise and travel, negatively impacted psychological health [28,29] but the long term consequences are unknown. The severe acute respiratory syndrome and middle-east respiratory syndrome epidemics have both previously demonstrated increases in psychiatric comorbidities such as depression, anxiety, panic attacks and suicidality [30,31,32].

During the initial onset of COVID-19, 25% of college students experienced symptoms of anxiety, whilst 53% of Chinese residents deemed the psychological impact of COVID-19 to be moderate or severe [33,34]. Recent research has identified reduced sleep quality during the COVID-19 outbreak as the primary mediator of negative emotions (e.g., stress and anxiety) during this time [32]. However, the authors did find that regular exercise could offset this [32]. There is little evidence concerning the effect of living through a pandemic or epidemic on menstrual function. This is partly due to the limited representation of female participants in research following these crises [35]. Despite this, one study conducted in Liberia following the 2014–2016 West African Ebola virus epidemic found that 27% women who had been eumenorrheic before the outbreak, experienced irregular menstruation afterwards [35]. However, as the Ebola virus also resulted in rapid weight loss, the authors note that it is unclear the specific reasons for which the irregularities occurred. One study did reveal the impact of the pandemic upon the menstrual cycle amongst a combined population of hormonal contraceptive pill users and eumenorrheic women [36]. The findings clearly highlighted that the changes in lifestyle associated with the pandemic, including alterations in exercise and eating behaviours led to alterations of the menstrual cycle length, symptoms, and/or in prevalence of menstrual dysfunction.

Particularly through the year 2020, COVID-19 resulted in significant disruption to daily life for individuals worldwide, with changes to work, social, diet, exercise patterns [37,38,39] and bringing with it increased amounts of stress [40,41,42]. Exercising women, who may already be at greater risk of menstrual dysfunction [43,44], when experiencing the heightened stress of COVID-19, may be particularly vulnerable to cycle length changes and/or adverse changes in symptomology. Given the importance of the menstrual cycle, and the known effects adverse symptoms can have on quality of life, the aim of this study was to investigate any changes in the pattern and symptoms of the menstrual cycle that exercising females may have experienced during the COVID-19 lockdown period, and to identify some of the main factors that contribute to these disturbances. Unlike previous studies, this sample will be restricted to those not utilising any form of hormonal contraception, given the known regularity to cycles that such interventions provide. The questionnaire will also capture detailed information on specific symptoms of the menstrual cycle and nutritional behaviours, that may or may not have changed within the pandemic. This will provide unique detail surrounding the pattern and symptoms of the menstrual cycle and the possible contributing influences through lifestyle changes associated with the pandemic.

## 2. Materials and Methods

### 2.1. Ethics and Participant Recruitment

The study was conducted in accordance with the declaration of Helsinki and approved by the institutional ethics committee at Nottingham Trent University. The survey was accessible online from 27 May 2020–17 June 2020. Advertisement of the study was carried out using social media channels and through local institutions of the researchers, all of which were approved through the ethical application process. Participants were eligible to if they were; aged ≥18 years; exercising at any level; eumenorrheic (having a pattern of at least 9 regular menstrual bleeds, over the previous 12 months, prior to COVID-19) without the use of a form of hormonal contraception. Participants from any country were able to complete the survey provided they met the above criteria.

### 2.2. Survey Development

The questionnaire contained 33 questions and was developed by all researchers, whom collectively have extensive experience with questionnaire design. The survey platform Qualtrics (Qualtrics 2005; 37, 892 ed., Provo, UT, USA) was used to make the questionnaire accessible online. The questionnaire was compiled of mainly binary or multiple-choice questions. The online link provided to the questionnaire began with the participant information sheet, followed by participant consent. By proceeding to the next stage participants were able to consent to the study. If participants did not wish to proceed, they were able to close the browser prior to consent. All participants were provided with a unique participant identification to ensure anonymity.

**Figure 1:**
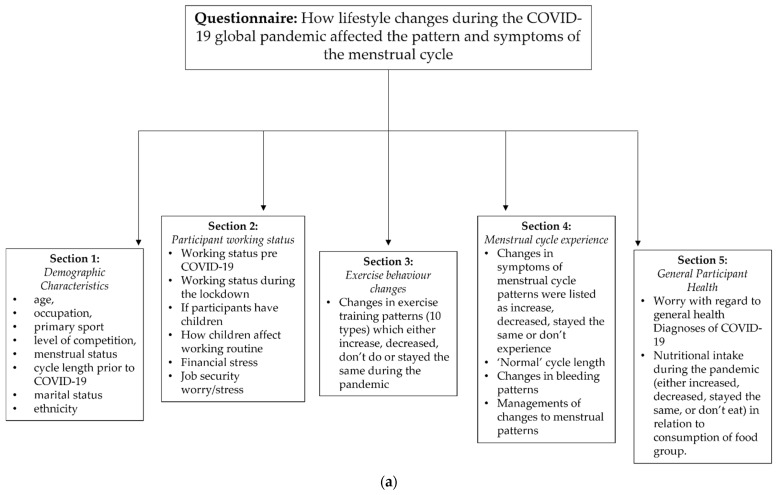

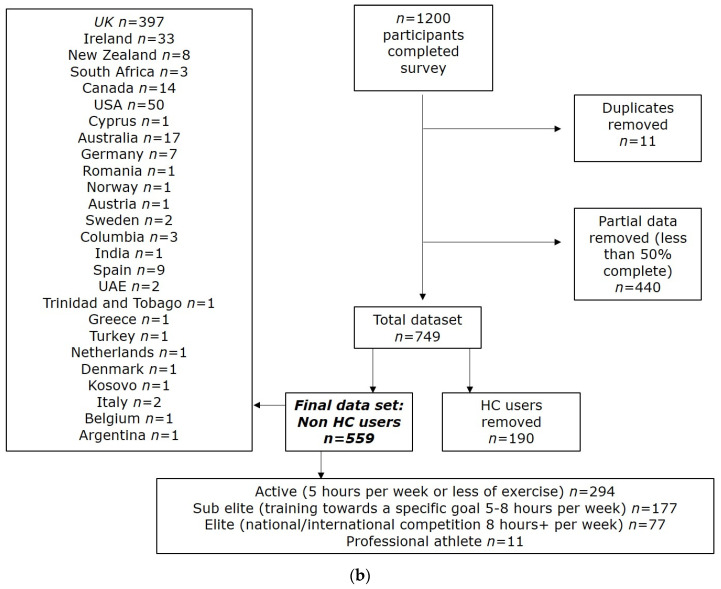
(**a**) Schematic representing the questionnaire design. (**b**). Data processing to achieve final data set (*n* = 559, non-hormonal contraceptive users).

The survey comprised of five sections. The first section provided an introduction and contained demographic related questions such as age, occupation, primary sport, level of competition, menstrual status, cycle length prior to COVID-19, marital status and ethnicity. The second section captured detail about the participants’ working status prior to COVID-19 and during the COVID-19 pandemic lockdown, information around children and if applicable, how this affected their working routine during the pandemic. This section also included Likert scales to enquire how financial status and job security affected worry and stress. The third section requested detail on changes to exercise behaviour that may or may not have occurred for the participant during lockdown. There were ten different categories of types of exercise training in each of which the participants could answer with one of the following options: 1; about the same, 2; increased, 3; decreased, 4; I never do this. All respondents were those who were exercising therefore no sedentary options were available within the questionnaire. The fourth section contained questions concerning the menstrual experiences of the participants, all symptoms that were asked about were based on previous work published by two authors [45,46]. This section also gathered information on the length of their cycle under normal circumstances, how their cycle changed during the pandemic, if applicable, whether their bleeding patterns have changed and if they have taken any action to manage any changes and/or symptoms. Similar options used in section three were applied here for 28 different symptoms associated with the menstrual cycle; participants stated if they had an increase in symptoms, no change, a decrease or did not regularly experience this symptom. Participants were also asked about the length of their cycle and bleeding patterns in a multiple-choice format (increased, decreased, stayed the same), with an opportunity to elaborate on coping mechanisms for any symptoms that may have arisen during the COVID-19 lockdown. The final section of the questionnaire focused on the general health of the participant. This included Likert scales regarding worry around personal and family health, and whether the participants have been previously diagnosed with, or have been assumed to have/had, COVID-19. The final element of the survey captured aspects of nutritional intake that may have changed during the lockdown using the previously mentioned scale (1–4) from Section 3 and Section 4. A schematic representing the questionnaire design is found in Figure 1a. Data processing is shown in Figure 1b.

### 2.3. Data Analysis

Data were analysed using IBM SPSS (v. 23.0 (IBM, Armonk, NY, USA)) and Microsoft Excel. Partial responses (<50%) were removed from the dataset. Data was coded to categorise responses and then frequency analysis was carried out. Data are represented as mean ± standard deviation (SD), or median (75th–25th quartiles of the interquartile range) as appropriate, frequencies and percentages. Binomial logistic regression was performed between those that have experienced changes in their menstrual cycle or not, to identify any significant alterations in exercise or nutritional behaviours between the two groupings. Following which Chi squared analysis was conducted, again between those that had experienced changes in their menstrual cycle and those that had not, to determine any significant differences in the prevalence of menstrual symptoms and experiences of stress, as well as differences between elite and active participants. Odds ratios were used to determine the likelihood of each symptom and stress parameter contributing to changes in cycle length. Statistical significance was set at *p* ≤ 0.05.

## 3. Results

### 3.1. Participant Characteristics

A total of 559 responses were included for final analysis. Participant characteristics and demographics are displayed in Table 1. Three of the participants had been diagnosed with COVID during the time of data collection. Fourteen percent of participants declared they had experienced some symptoms of COVID-19 and therefore had to self-isolate. Twelve percent reported that someone in their household had experienced symptoms and in turn they were required to self-isolate.

### 3.2. Participant Stress and Employment

Prior to COVID-19, 73% of our sample were in full-time employment. During COVID-19 lockdown half of the respondents reported to be working part-time at home and 18% were working full time at home. Only 34 participants indicated they had been furloughed at either 80% or 100% pay. Distribution of working status prior to and during COVID-19 is shown in Table 1.

The stress experienced by participants is displayed in Table 2. The majority of those with children expressed that their children caused higher levels of daily stress. When considering other possible sources of stress and worry such as financial stress, job security and stress regarding menstrual symptoms, most participants (>50%) did not report being overly worried or stressed (1–3 on Likert scale) by such parameters (Table 2).

### 3.3. Menstrual Cycle Characteristics and Symptoms

Prior to the onset of the first COVID-19 lockdown the median cycle length was 28 days (30–26 days). During the pandemic, at the time of data collection, 295 (52.8%) participants reported to have experienced a change in the length of their menstrual cycle. One participant reported to have had a total absence of bleeding, whilst another reported to have experienced continuous bleeding. There was a median increase in cycle length reported of 5 days (minimum 2 days, maximum 32 days), with a median decrease of 3 days (minimum 2 days and maximum 17 days). In the section of the questionnaire, where free text was allowed, a number of participants reported to have experienced changes in cycle length that varied somewhat. Some examples are:

“Really random have had one week between periods then two”

“More stable and regular. Was 28–40 day cycles. Now 29 days”

“Two cycles were increased (longer), the third cycle decreased (shorter than normal)”

“Only 3 days break before bleeding again”

“Become more irregular”

A large proportion of participants (42%) reported a decrease in the bleeding time during their menstrual cycle, with just 8% reporting an increase. Of those that reported an increase the average number of days bleeding time was reported to increase was 5 with a median of 3 days (minimum of 2 and maximum of 16). The average number of days bleeding time was reported to decrease by was 2.25 days, with a median of 2 days (minimum 1 day less and maximum of 10 day decrease). Similarly, a number of participants chose to report details of their bleeding pattern changes. Some of which were as follows:

“My period seems out of sorts”

“No period”

“Very heavy at the start then rapid taper off”

“Period has become irregular”

**Figure 2:**
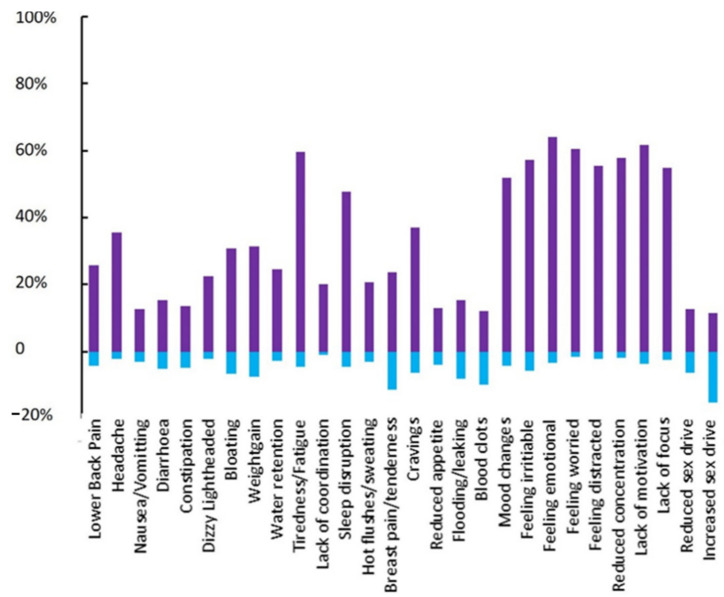
Menstrual Cycle symptoms reported by eumenorrheic participants (*n* = 559). This Figure represents the percentage of participants reporting symptoms that have increased (purple) and decreased (blue).

**Figure 3:**
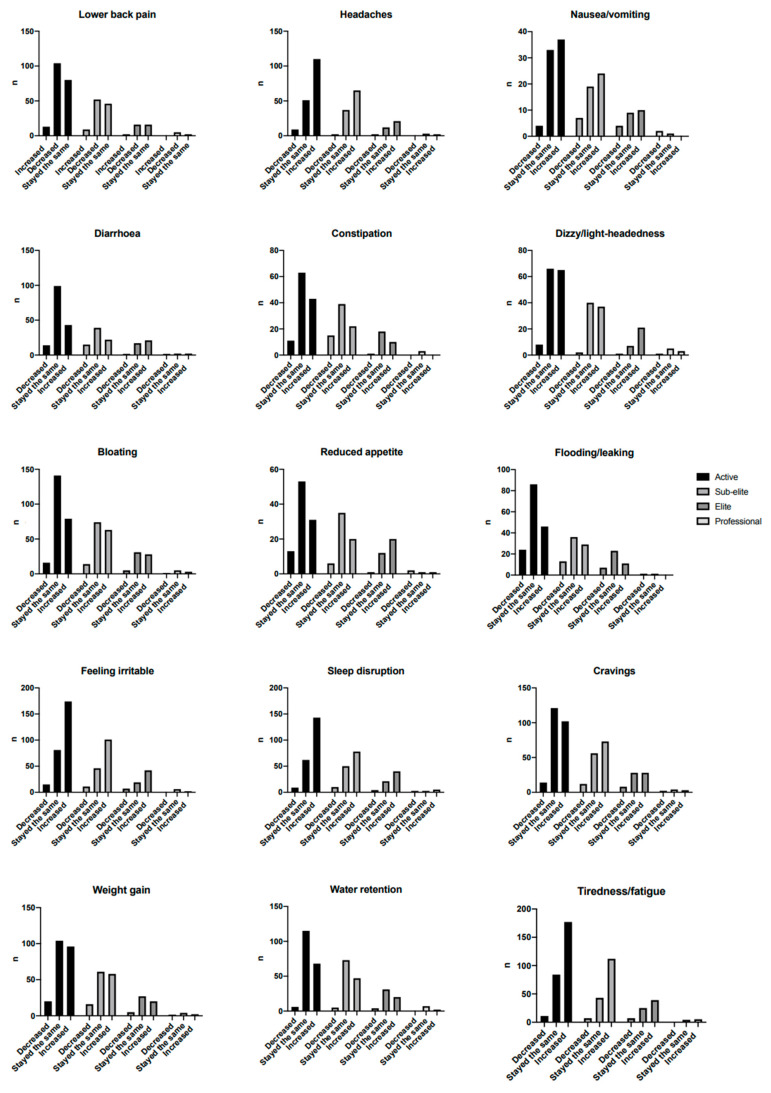

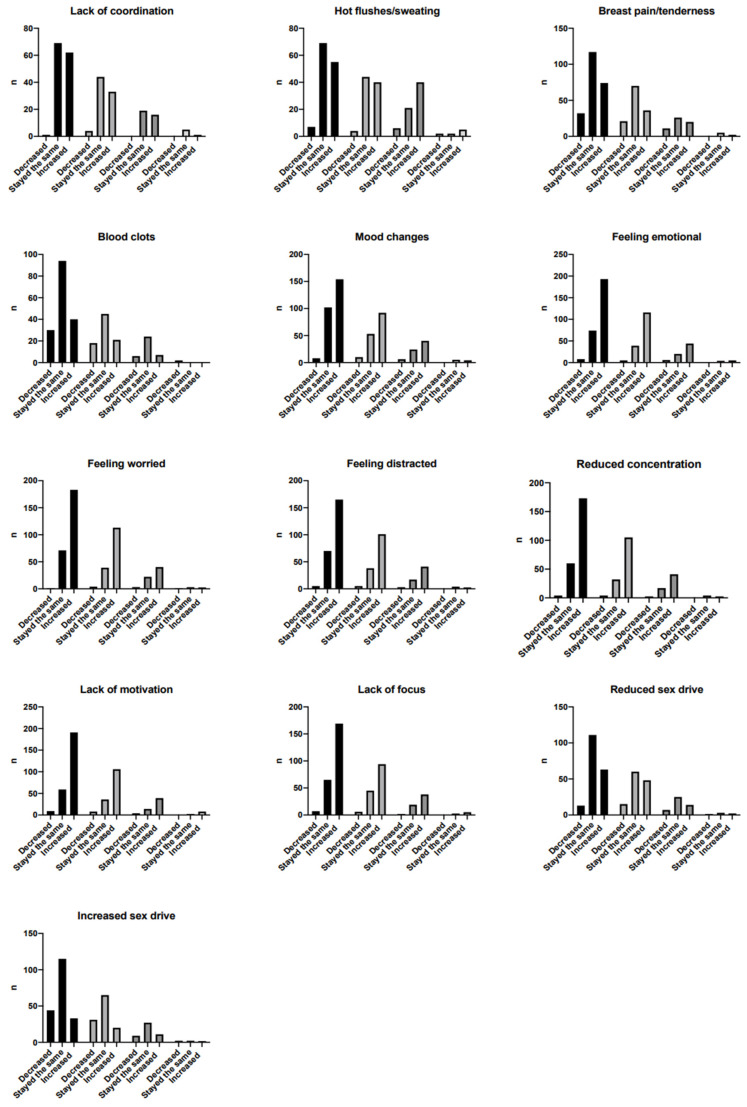
Changes in all Menstrual cycle symptoms that have been reported, according to level of activity. Significant (*p* < 0.05) difference between groups were identified for; Reduced appetite (active vs. elite *p* = 0.010, sub elite vs. elite *p* = 0.014), feeling irritable (active vs. professional *p* = 0.002, sub elite vs. professional *p* = 0.003, elite vs. professional *p* = 0.003) and feeling worried (active vs. professional *p* = 0.018, sub elite vs. professional *p* = 0.010). n; refers to the frequency of responses.

Most changes to menstrual *symptoms* experienced during the COVID-19 pandemic were psychosocial in nature (Figure 2 and Figure 3). Over half of all participants reported to have experienced lack of motivation (61.5%), focus (54.7%) and concentration (57.8%). Similarly, symptoms of mood changes, irritability, emotional feeling, worry and being distracted were all reported to have increased during the pandemic in over half of all participants. Figure 3 displays all reported symptoms that have increased, decreased (A) stayed the same or not been experienced (B). Reported symptoms were also divided between activity status. Significant differences were identified, through one way ANOVA, in reduction in appetite, feeling irritable and feeling worried as detailed in Table 3.

A variety of participants chose to provide comments with regard to their changes in symptoms during this time period:

“….I’ve noticed an increase in pre menstrual symptoms and feeling very emotional while in my period”

“More cramping, earlier cramping, more luteal phase spotting”

“…..my lymphatic nodes/glands have been unusually swollen and tender so, armpits, groin, back of head, nape of neck while actually menstruating, they’ve gone down when I’ve stopped bleeding”

“I’m getting yeast infections after my period—twice now. That used to be extremely rare”.

### 3.4. Nutrition and Exercise Patterns

Changes in nutritional and exercise patterns can be found in Table 4. Changes to patterns in exercise aligned with the worldwide restrictions that were in place during the pandemic. Sport specific skill training was reported to have decreased by 36% of participants, whilst increases were reported in aerobic endurance training (40%), body weight conditioning (43%) and core stability exercises (43%). The majority of eating behaviours remained consistent; however, it is worth noting that there were increases reported in the consumption of less healthy foods (46%), home baked goods (68%) and alcohol (40%).

Binary logistic regression and Chi squared analysis was performed to determine any significant associations of the behaviour patterns and symptoms reported by participants. All data of which can be found in Supplementary Tables S1–S5. From the data collated, exercise patterns (Supplementary Table S1) and stress (Supplementary Tables S4 and S5) did not significantly contribute to changes in menstrual cycle length. A lack of focus was significantly associated with a change in menstrual cycle length (*p* = 0.038) reported to have increased by 61% of participants, other symptoms were not significantly associated (Supplementary Table S3) with changes in menstrual cycle patterns. Finally, changes to eating patterns of white meat (increase *p* = 0.035, decrease *p* = 0003) and processed meat (increase *p* = 0.002 and decrease *p* = 0.001) were significantly associated with a change in menstrual cycle length (Supplementary Table S2).

## 4. Discussion

The purpose of this research was to firstly identify how lifestyle changes within the first lockdown of the COVID-19 pandemic have affected the pattern and symptoms of the menstrual cycle amongst eumenorrheic females. Five hundred and fifty-nine participant responses were included for final analysis, with 49.7% citing a change in their menstrual cycle length. Over two thirds (70.8%) of participants experienced a change in bleeding patterns. Secondly, the study aimed to identify the main contributing factors to such changes in the menstrual cycle through questions around dietary, exercise and stress behaviours. Changes (either an increase or decrease) in length of the menstrual cycle were significantly associated with eating both white meat and processed meats, whilst eating less healthy foods over the pandemic was associated with a decrease in cycle length. Despite numerous symptoms of the menstrual cycle were reported to be experienced, a lack of focus was the only symptom to be associated with changes in cycle length. Surprisingly, higher stress was not associated with a greater likelihood of a change in cycle length despite over 50% of the participants reporting high stress in relation to worry about their families’ health. Stress is known to elicit changes to the hypothalamic-pituitary-gonadal axis which causes the dysregulation of gonadotropin releasing hormone secretion, often accompanied by heightened levels of cortisol [47,48,49]. Phelan et al. [36] conducted a similar research project during the first 6 months of the pandemic, in which significant associations between stress and menstrual cycle disturbances, were identified. Clearly, the climate during the initial onset of COVID-19, and subsequent lockdowns, was a stressful event for many [50,51,52]. This data highlights the great impact that such stress can have on female physiology, not solely on mental health and well-being, and how alterations in behaviour patterns can influence the usual patterns of the menstrual cycle. Current education around such possible influencers on the menstrual cycle is limited amongst the female population [53,54]. Given that the uncertainty of COVID-19 has continued for ~2 years and is likely to still be an ongoing part of the environment for the foreseeable future, it is important that females become aware of the influence that stress will have on the menstrual cycle and are able to manage/control these changes to the best of their ability, while also having an appreciation of the potential use of changes to their menstrual cycle to indicate a cause for concern. Despite no significant findings with regard to stress within this current study, it is deemed that long-term stress could have a negative impact on fertility and female reproductive health, [55,56,57], and is also suggested that exacerbated stress can induce premature brain ageing [58]. Therefore, it is especially important for females of childbearing age, and clinicians alike, to consider the possible factors, including stress, that could be contributing to any presenting changes in menstrual cycle characteristics. Given that our data still highlighted high levels of stress, combined with significant associations documented elsewhere [36], it is certainly deemed that the initial pandemic has caused stress for a number of women. Our lack of significant findings in relation to stress could be attributed to slightly smaller participant numbers or given our sample population was specifically exercising females, the exercise may have offset some of the levels of stress experienced by some of the females answering the questionnaire. It is already known that exercise and a balanced sound nutritional intake are often recommended to off-set some of the heightened stress [59,60,61,62].

Most menstrual cycle-associated symptoms that showed evidence of change during the initial onset of the COVID-19 pandemic, and subsequent lockdown, were psychosocial in nature. Over half of all participants reported a change in mood (54%), irritability (59.9%), emotional feeling (66.5%), worry (61.0%), feeling distracted (56.6%), lack of concentration (56.9%), motivation (62.3%) and focus (55.7%). Again, these results aligned with Phelan et al. [36], who identified 84% of participants reporting at least one mental health symptom during the pandemic.

There were some differences in the menstrual cycle symptoms reported between elite and sub-elite athletes, compared to non-elite respondents. For active women a change in cycle length was significantly associated with a lack of focus, whilst for elite activity level women, lower back pain and a lack of motivation were associated with a change in cycle length. Mood changes are one of the major symptoms that have been reported to have been experienced by active women [46], in contrast to elite athletes who tend to report more physical symptoms such as stomach cramps and back pain [45]. The reporting of a lack of motivation by elite athletes is not surprising given there was a huge decline in competition and training availability throughout the start of the pandemic, which would certainly be a factor in motivation levels for these athletes [63]. Within our sample it seems that changes in exercise behaviours were not associated with any changes in menstrual cycle length, as detailed in Supplementary Table S1. Given our sample consisted of those who were at least physically active, both before and during the pandemic, there have not been any dramatic changes in physical activity, thus it is not surprising these patterns were not associated with changes in menstrual cycle length. It appears that the first COVID-19 lockdown had a greater impact on the non-physical symptoms of the menstrual cycle in active women. The importance of psychosocial symptoms surrounding the menstrual cycle should not be neglected and it is important that such symptoms are captured by females and clinicians/practitioners when collating information in relation to the menstrual cycle. The impact of the pandemic on mental health has also been widely reported; [64] psychosocial symptoms surrounding the menstrual cycle, if left untreated and the female continues to suffer and experience such symptoms, are likely to cause a decline in mental health as COVID-19 continues to be a profound presence.

Influences of nutritional intake and exercise on the menstrual cycle have been well documented [65,66,67,68]. During these 24 months, across the globe, varying degrees of lockdown arose, inevitably affecting the ability of many females to carry out exercise. During the first months of the pandemic the majority of sporting facilities were closed, and in some countries, restrictions limited people to within a 1 km radius from their homes. Inevitably, nutritional habits changed in this timeframe [69,70,71], which is also supported by our data. Not unexpectedly, participants reported to have increased alcohol intake, consumption of cooked and baked goods and generally increased consumption of unhealthy foods. This is not unsurprising given the evidence of comfort eating during periods of heightened stress [72,73], and the potential for supply chain issues with fresh food. Interestingly, an increase in dairy consumption was associated with cycle length increases. Previous studies have provided reports that support the possibility of dairy contributing to anovulatory cycles, with increases in length of the luteal phase. Over consumption of dairy products, in the long term, could result in more permanent changes in reproductive hormones and long-term fertility issues [74,75]. Continual changes to dietary habits could contribute to longer term reproductive issues, although such issues are of course multifactorial in nature, it should not be dismissed that the environment of pandemics could impact reproductive function in-directly.

Changes to meat consumption, both increases and decreases, were significantly associated with changes to the menstrual cycle length during the initial pandemic. Whilst this data does not provide a succinct result, this information aligns with the literature. Some reports have suggested that there are more likely to be increases in menstrual cycle length with meat diets over non-meat products such as soya [76], whilst other research indicates that meat eaters have a greater regularity of menstrual cycles over vegetarians [77] and vegetarians are less likely to experience anovulatory disturbances compared to non-vegetarians [78]. It is speculated that there could be some contribution to cycle irregularity amongst vegetarians and meat eaters, however, exploring this possibility was beyond the scope of this manuscript. Long term monitoring of menstrual cycle patterns and/or symptoms are needed to further conclude on the impact of meat consumption and dietary behaviours on this aspect of female physiology.

This current study is one of the few studies [36] to detail the implications of the initial pandemic on the menstrual cycle patterns and symptoms of females along with their associated psychosocial symptoms, physical activity and nutritional behaviours. The data provided herein not only highlights the potential influence of surrounding environmental stressors and changes in behaviours in relation to maintaining a consistent menstrual cycle, but also provides important detail for practitioners/clinicians when managing patients and athletes with irregularities of the menstrual cycle. Given that the onset of the pandemic was a unique experience during its time, it is important that later research and clinical practice addresses any long-term implications of such environmental changes.

As with all research, there are limitations with this study that should be acknowledged. As with the majority of questionnaire-based research, researchers are reliant upon true responses from the participants and accuracy when recall is required. Some previous studies have demonstrated measurement error when using self-report data regarding the menstrual cycle [79]. Additionally, there could be an element of bias within our sample, with those who have changes to their menstrual cycle symptoms during the pandemic more likely to have complete the survey. However, given the advantages that digital surveys allow such as large reach of participants and quick data collection in an appropriate anonymous format, and that data collection was during a time of restricted travel and physical interaction, the findings of our study still provide a very useful insight into the impact of the lockdown on menstrual cycle patterns and symptoms. The data herein also comes from a range of countries in which there were a range of rules and regulations, and therefore experiences, with regard to lockdown, possibly influencing the participants differently. However, the countries assessed in the study all documented some degree of change in lifestyles and working environments. Our data did not capture information on smoking status or body mass index (BMI). Smoking patterns appear to fluctuate during different phases of the menstrual cycle [80], thus if females experience an increase in cycle symptoms or a change in patterns, this in turn could influence their smoking behaviours. Additionally, smoking status can influence menstrual cycle irregularity, thus it would be an important element to capture [4]. Similarly with BMI, at either end of the spectrum, there are known associations with menstrual irregularities, thus a possible contributing factor to menstrual cycle symptoms and changes in characteristics [81,82], especially given the possible fluctuations in eating behaviours and weight throughout the pandemic [36]. Finally, our data only provides a small insight into the changes experienced by females during the initial onset of COVID-19 pandemic and subsequent lockdown; it does not provide any longitudinal data that may document the rise and fall of symptom exacerbation or the degree of severity that the females may have been affected by such environmental changes.

## 5. Conclusions

In conclusion, lifestyle changes experienced by females throughout the initial COVID-19 pandemic lockdown period can impact menstrual cycle length and symptoms. Over half of all females reported a change in their cycle length and over a third experienced changes in bleeding patterns. An average of 34% reported an increase in at least one symptom. The most substantial changes arose in psychosocial symptoms, which is somewhat expected given previously reported links between the pandemic lockdown period and mental health. This is important data and highlights the potential for long-term stress to influence female fertility and other health consequences. With the ongoing uncertainty of COVID-19, and continued peaks and troughs on the number of COVID-19 cases, continued menstrual cycle changes could become a serious consequence and impact long-term fertility. It is therefore important to educate females about the possible influence of environmental stress on their physiology, as well as to ensure clinicians are fully aware of the reasons why menstrual cycle changes may arise.

Finally, our data provide further reasoning as to why the menstrual cycle should become a pivotal monitoring tool for all females to indicate stability, a practice that would be recommended to all females from our current findings. Tracking the menstrual cycle is a relatively simple practice that will allow females to understand the wide range of symptoms associated with the menstrual cycle, and readily identify alterations that may arise alongside life stressors. Given the menstrual cycle can be altered by a wide range of factors, such as; stress, anxiety, and nutrition, it has been recommended to follow best practice for each individual factor, collectively, in order to prevent any long term changes to the pattern of the menstrual cycle [83]. If changes are experienced, then a holistic approach is required with regard to treatment, taking into consideration the abundance of possible influencing factors.

## Figures and Tables

**Table 1:** Participant characteristics and demographics (*n* = 559).

Characteristic	Categories	*n*	%
Age (years)	15–20	30	5.5
	21–30	200	35.7
	31–40	199	35.6
	41–50	122	21.8
	51+	7	1.4
Mean Age 33.24 ± 8.38 yrs			
Activity level	Active/fitness training (5 h or less per week)	294	52.5
	Sub elite training (training towards a goal, 5–8 h per week)	177	31.7
	Elite training (national/international competition, 8 h + per week)	77	13.8
	Professional Athlete	11	2.0
Marital Status	Divorced	8	1.4
	Living with partner	122	21.8
	Single	199	35.6
	Married	212	38.0
	Widowed	2	0.4
	Other	16	2.8
Ethnicity	Asian/Asian Black	7	1.30
	Black/African/Caribbean/Black British	4	0.72
	Mixed/Multiple ethnicity	19	3.40
	White	525	94.0
	Other	2	0.35
	Rather not say	2	0.35
Menstrual cycle length prior to COVID-19	21 days or less	50	9.0
	22–35 days	477	85.3
	36–89 days	22	4.9
	90+ days	4	0.8
Prior to COVID-19 lock down March 2020 were you working/studying	Full time	409	73.2
	Part time	100	17.9
	Unemployed	13	2.3
	Carer	2	0.3
	Full time Athlete	7	1.3
	Other	28	5.00
During the pandemic/lockdown 1 how were you working	Part time at home	279	49.9
	Full time at home	102	18.2
	Furlough at 100% pay	9	1.6
	Furlough at 80% pay	25	4.5
	Other	144	25.8
Have you been diagnosed with COVID	Yes	3	0.5
	No	555	99.5
Have you had symptoms and suspected you had COVID	No	475	85.0
	Yes	83	14.8
Has anyone in your household had symptoms and therefore you have isolated	No	491	87.8
	Yes	67	12.0

**Table 2:** Likert stress scale responses from the online survey taken during the initial onset of the global pandemic, COVID-19. (1 = the least stressed and 7 = extremely stressed).

Question	Likert Scale	Frequency (*n*)	Percentage (%)
Do you have children?	Yes	152	27.2
	No	407	72.8
Do your children affect your normal routine during COVID	Yes	110	72.4
	No	42	27.6
For those with children; Please highlight how your children have affected your daily stress levels during the lock down.	1	10	6.6
	2	14	9.2
	3	29	19.1
	4	37	24.3
	5	46	30.3
	6	16	10.5
	7	8	5.3
Please highlight how worried you are about job security during COVID-19 and moving forward from here.	1	236	42.2
	2	194	34.7
	3	66	11.8
	4	53	9.5
	5	48	8.6
	6	32	5.7
	7	29	5.2
Please highlight how Worried you are about personal finances during the COVID-19 pandemic.	1	178	31.8
	2	160	28.6
	3	86	15.4
	4	68	12.2
	5	48	8.6
	6	33	5.9
	7	28	5.0
If your cycle length has changed please highlight how stressed you are about these changes	1	152	27.2
	2	88	15.7
	3	69	12.3
	4	49	8.8
	5	39	7.0
	6	21	3.8
	7	14	2.5
Please highlight how worried you are about your own health during the current pandemic	1	122	21.8
	2	124	22.2
	3	110	19.7
	4	81	14.5
	5	67	12.0
	6	25	4.5
	7	17	3.0
Please highlight how worried you are about your families health during the current pandemic.	1	31	5.5
	2	62	11.1
	3	89	15.9
	4	84	15.0
	5	116	20.8
	6	87	15.6
	7	83	14.8

Likert scales 1–7 (7 being extremely likely and 1 being least likely).

**Table 3:** Exercise behaviour changes for all participants during the initial phases of COVID-19 pandemic.

Exercise	Answer	Frequency	Percentage %
Sports-specific skill training (ie practice of skills associated with your sport)	Don’t do this	99	17.7
	Decreased	205	36.7
	Stayed the same	125	22.4
	Increased	129	23.1
Aerobic endurance training (i.e., continuous running/cycling/rowing)	Don’t do this	40	7.2
	Decreased	157	28.1
	Stayed the same	138	24.7
	Increased	223	39.9
Aerobic-based interval training (i.e., repetitions > 2 min duration, short recoveries)	Don’t do this	117	20.9
	Decreased	158	28.3
	Stayed the same	148	26.5
	Increased	134	24.0
High-intensity interval and sprint training (i.e., repetitions < 2 min duration, long recoveries)	Don’t do this	138	24.7
	Decreased	168	30.1
	Stayed the same	135	24.2
	Increased	116	20.8
Multi-joint resistance training (i.e., barbell, dumbell, kettlebell, machine exercises)	Don’t do this	144	25.8
	Decreased	213	38.1
	Stayed the same	100	17.9
	Increased	100	17.9
Single-joint resistance training (e.g., calf raises, theraband exercises, lateral raise)	Don’t do this	150	26.8
	Decreased	125	22.4
	Stayed the same	126	22.5
	Increased	156	27.9
Plyometrics (i.e., jumping, hopping, skipping, bounding)	Don’t do this	216	38.6
	Decreased	117	20.9
	Stayed the same	94	16.8
	Increased	130	23.3
Body weight conditioning (e.g., press-ups, burpees, high knees, star jumps)	Don’t do this	92	16.5
	Decreased	96	17.2
	Stayed the same	128	22.9
	Increased	241	43.1
Core stability/pilates (exercises specifically for the trunk/abdominal region)	Don’t do this	66	11.8
	Decreased	101	18.1
	Stayed the same	150	26.8
	Increased	240	42.9
Static stretching	Don’t do this	83	14.8
	Decreased	100	17.9
	Stayed the same	205	36.7
	Increased	169	30.2

**Table 4:** Nutritional behaviour changes for all participants during the initial phases of COVID-19 pandemic.

Nutritional Food Group	Answer	Frequency	Percentage %
Eating fruits and vegetables	Don’t eat this	0	0.0
	Decreased	90	16.1
	Stayed the same	295	52.8
	Increased	171	30.6
Eating dairy products	Don’t eat this	50	8.9
	Decreased	57	10.2
	Stayed the same	317	56.7
	Increased	133	23.8
Eating red meat e.g., beef, lamb, pork (protein)	Don’t eat this	136	24.3
	Decreased	72	12.9
	Stayed the same	258	46.2
	Increased	91	16.3
Eating white meat e.g., chicken, turkey	Don’t eat this	115	20.6
	Decreased	55	9.8
	Stayed the same	306	54.7
	Increased	81	14.5
Eating processed meat e.g., bacon, sausages, ham	Don’t eat this	189	33.8
	Decreased	86	15.4
	Stayed the same	196	35.1
	Increased	86	15.4
Eating fish	Don’t eat this	110	19.7
	Decreased	84	15.0
	Stayed the same	249	44.5
	Increased	114	20.4
Eating grains/carbohydrates (for example rice, pasta, potatoes)	Don’t eat this	5	0.9
	Decreased	48	8.6
	Stayed the same	329	58.9
	Increased	174	31.1
Eating healthy fats (nuts, seeds, avocados, nut butters, oils)	Don’t eat this	22	3.9
	Decreased	50	8.9
	Stayed the same	366	65.5
	Increased	119	21.3
Eating less healthy/processed foods (cakes, sweets, biscuits, chocolates)	Don’t eat this	28	5.0
	Decreased	100	17.9
	Stayed the same	172	30.8
	Increased	257	46.0
Eating more home baked/cooked foods	Don’t eat this	16	2.9
	Decreased	14	2.5
	Stayed the same	145	25.9
	Increased	382	68.3
Eating more take outs	Don’t eat this	95	17.0
	Decreased	279	49.9
	Stayed the same	126	22.5
	Increased	57	10.2
Drinking alcohol	Don’t eat this	99	17.7
	Decreased	113	20.2
	Stayed the same	116	20.8
	Increased	229	41.0

## Data Availability

The datasets generated and analysed during the current study are available from the corresponding author upon reasonable request.

## Supplementary Materials

The following supporting information can be downloaded at: https://www.mdpi.com/article/10.3390/ijerph192013622/s1, Table S1: Binary logistic regression for those participants who reported a change in their menstrual cycle length (exercise behaviours); Table S2: Binary logistic regression for those participants who reported a change in their menstrual cycle (dietary beahviours); Table S3: Chi Square analysis to determine any significant difference in participants who reported a change in cycle length between a change in menstrual cycle symptoms; Table S4: Chi Square analysis to determine any significant difference in participants who reported a change in cycle length and a high/low stress; Table S5: Chi Square analysis to determine any significant difference in participants who reported a change in cycle length between a change in menstrual cycle symptoms (Elite and Active).
