# Supplementary material for: How Lifestyle Changes during the COVID-19 Global Pandemic Affected the Pattern and Symptoms of the Menstrual Cycle
Source: Int J Environ Res Public Health. 2022 Oct 20;19(20):13622. doi: 10.3390/ijerph192013622 (PMC9602509; doi:10.3390/ijerph192013622)
Supplement: Supplementary file 1 [file ijerph-19-13622-s001.zip › ijerph-1958359-supplementary.pdf]

Supplementary Table S1. Binary logistic regression for those participants who reported a change in their menstrual cycle length (n=295) to determine any associations with an increase or decrease in exercise behaviours

Exercise	Answer	P Value	Odds ratio	95% CI Lower	95% CI Upper
Sports-specific skill training (ie practice of skills associated with your sport)	Decreased	0.354	0.735	0.383	1.410
	Increased	0.200	0.669	0.362	1.237
Aerobic endurance training (i.e. continuous running/cycling/rowing)	Decreased	0.095	0.482	0.205	1.135
	Increased	0.589	1.159	0.678	1.980
Aerobic-based interval training (i.e. repetitions >2 min duration, short recoveries)	Decreased	0.789	0.915	0.476	1.757
	Increased	0.755	0.904	0.480	1.703
High-intensity interval and sprint training (i.e. repetitions <2 min duration, long recoveries)	Decreased	0.793	0.916	0.474	1.768
	Increased	0.654	1.170	0.588	2.328
Multi-joint resistance training (i.e. barbell, dumbell, kettlebell, machine exercises)	Decreased	0.822	1.083	0.543	2.159
	Increased	0.122	0.558	0.267	1.169
Single-joint resistance training (e.g. calf raises, theraband exercises, lateral raise)	Decreased	0.654	0.862	0.450	1.652
	Increased	0.996	0.766	0.391	1.497
Plyometrics (i.e. jumping, hopping, skipping, bounding)	Decreased	0.622	0.863	0.481	1.549
	Increased	0.988	0.995	0.507	1.952

Body weight conditioning (e.g. press-ups, burpees, high knees, star jumps)	Decreased	0.201	1.624	0.772	3.415
	Increased	0.345	1.356	0.720	2.555
Core stability/Pilates (exercises specifically for the trunk/abdominal region)	Decreased	0.875	0.942	0.444	1.996
	Increased	0.470	0.815	0.469	1.418
Static stretching	Decreased	0.460	0.785	0.414	1.490
	Increased	0.723	0.917	0.566	1.484

Supplementary Table S2. Binary logistic regression for those participants who reported a change in their menstrual cycle length (n=295) to determine any associations with an increase or decrease in dietary behaviours

Exercise	Answer	P Value	Odds ratio	95% CI Lower	95% CI Upper
Eating fruits and vegetables	Decreased	0.097	4.387	0.766	25.154
	Increased	0.901	1.075	0.342	3.380
Eating dairy products	Decreased	0.730	1.440	0.182	11.401
	Increased	**0.025**	0.936	0.238	3.676
Eating red meat e.g. beef, lamb, pork (protein)	Decreased	0.085	0.076	0.004	1.425
	Increased	0.477	1.810	0.353	9.280
Eating white meat e.g. chicken, turkey	Decreased	**0.003**	160.632	5.553	4646.444
	Increased	**0.035**	16.473	2.342	115.876
Eating processed meat e.g. bacon, sausages, ham	Decreased	**0.001**	43.038	4.301	430.621
	Increased	**0.002**	13.702	2.614	71.837
Eating fish	Decreased	0.153	0.255	0.039	1.664
	Increased	0.499	0.588	0.126	2.745
Eating grains/carbohydrates (for example rice, pasta, potatoes)	Decreased	1.00	0.0	0.0	0.0
	Increased	0.028	0.232	0.063	0.852
Eating healthy fats (nuts, seeds, avocados, nut butters, oils)	Decreased	0.430	0.103	0.000	28.959
	Increased	0.906	1.081	0.294	3.970
Eating less healthy/processed foods	Decreased	**0.035**	0.032	0.001	0.779
	Increased	0.124	0.376	0.108	1.306

(cakes, sweets, biscuits, chocolates)					
Eating more home baked/cooked foods	Decreased<br><br>Increased	0.583<br><br>0.321	0.439<br><br>0.545	0.023<br><br>0.164	8.332<br><br>1.807
Eating more take outs	Decreased<br><br>Increased	0.678<br><br>0971	1.555<br><br>0.967	0.193<br><br>0.161	12.515<br><br>5.812
Drinking Alcohol	Decreased<br><br>Increased	0.953<br><br>0.084	0.956<br><br>0.303	0.217<br><br>0.078	4.206<br><br>1.176

Supplementary Table S3. Chi Square analysis to determine any significant difference in participants who reported a change in cycle length between a change in menstrual cycle symptoms. Odds ratio to determine the likelihood of an increase in symptom causing a change in menstrual cycle length

Symptom	Change in Symptom (n/%)	No change in Symptom (n/%)	Chi Squared	P value	Odds ratio	95% Confidence Intervals	
						Lower	Upper
Lower Back pain	210/71.9	85/28.1	0.457	0.499	0.916	0.712	1.180
Headaches	114/38.6	181/61.4	0.214	0.643	1.052	0.849	1.303
Nausea/Vomiting	47/15.9	248/84.1	0.117	0.896	1.026	0.698	1.507
Diarrhoea	62/21.0	233/79.0	0.076	0.783	1.047	0.755	1.452
Constipation	54/18.3	241/81.7	0001	0.970	1.007	0.708	1.431
Dizzy/light headed	76/25.8	219/74.2	0.531	0.466	1.115	0.832	1.495
Bloating	110/37.3	185/62.7	0..021	0.886	0.984	0.795	1.219
Weight gain	113/38.3	182/61.7	0.126	0.722	0.963	0.783	1.185
Water retention	81/27.5	214/72.5	0.022	0.881	1.021	0.778	1.340
Tiredness/Fatigue	188/63.7	107/36.3	0.066	0.797	0.984	0.869	1.114
Lack of coordination	64/21.7	231/78.3	0.221	0.639	1.081	0.782	1.494
Sleep disruption	149/50.5	146/49.5	1.648	0.199	0.901	0.769	1.056
Hot flushes/sweating	70/23.7	225/76.3	0.005	0.946	1.010	0.749	1.362

Breast pain/tenderness	106/35.9	189/64.1	0.207	0.649	1.054	0.840	1.322
Cravings	129/43.7	166/56.3	0.049	0.825	1.022	0.845	1.236
Reduced appetite	241/81.7	54/18.3	0.991	0.320	1.208	0.831	1.755
Flooding/leaking	76/25.7	219/74.3	1.887	0.170	1.237	0.912	1.677
Blood clots	226/76.6	69/23.4	0.527	0.468	1.123	0.821	1.535
Mood changes	170/57.6	125/42.4	0.537	0.464	1.056	0.912	1.224
Feeling irritable	188/63.7	107/36.3	0.154	0.694	1.026	0.903	1.166
Feeling emotional	205/69.5	90/30.5	1.195	0.274	1.067	0.950	1.198
Feeling worried	188/63.7	107/36.3	0.726	0.394	1.058	0.929	1.206
Feeling distracted	174/54.0	121/46.0	0.487	0.485	1.052	0.912	1.214
Reduced concentration	184/62.4	111/37.6	1.787	1.181	1.092	0957	1.260
Lack of motivation	202/68.5	93/31.5	2.786	0.095	1.109	0.981	1.254
Lack of focus	181/61.4	114/38.6	4.313	**0.038**	1.165	1.007	1.348
Reduced sex drive	88/29.8	207/70.2	0.136	0.712	1.050	0.810	1.361
Increased sex drive	80/27.1	215/72.9	0.004	0.952	1.008	0.767	1.325

Supplementary Table S4. Chi Square analysis to determine any significant difference in participants who reported a change in cycle length and a high stress (likert scale 5-7) and low stress (likert scale 1-4). Odds ratio to determine the likelihood of a high stress causing a change in menstrual cycle length

Symptom	High stress (n/%)	Low stress (n/%)	Chi Squared	P value	Odds ratio	95% Confidence Intervals	
						Lower	Upper
Please highlight how worries you are about job security during COVID-19 and moving forward from here	57/19.3	238/80.7	0.012	0.911	0.981	0.700	1.374
Please highlight how worried you	65/22.0	230/78.0	2.557	0.110	1.322	0.937	1.866

are about personal finances during the COVID-19 pandemic							
If your cycle length has changed please highlight how stressed you are about these changes	40.0/13.6	255/86.4	0.055	0.813	1.053	0.688	1.612
Please highlight how worried you are about your own health during the current pandemic	57/19.3	238/80.7	0.012	0.911	0.981	0.700	1.374
Please highlight how worried you are about your families health during the current pandemic	152/51.5	143/48.5	0.033	0.856	1.015	0.863	1.194

Supplementary Table S5. Chi Square analysis to determine any significant difference in participants who reported a change in cycle length between a change in menstrual cycle symptoms. Odds ratio to determine the likelihood of an increase in symptom causing a change in menstrual cycle length. Active (A) and Elite (B) Participants.

A

Symptom	Change in Symptom (n/%)	No change in Symptom (n/%)	Chi Squared	P value	Odds ratio	95% Confidence Intervals	
						Lower	Upper
Lower Back pain	78/31.8	167/68.2	0.041	0.840	1.028	0.787	1.343
Headaches	101/41.2	144/58.8	0.643	0.423	1.096	0.875	1.372
Nausea/Vomiting	39/15.9	206/84.1	0.157	0.692	1.090	0.711	1.671
Diarrhoea	52/21.2	193/78.8	0.223	0.637	1.090	0.762	1.560
Constipation	48/19.6	197/80.4	0.024	0.877	1.0303	0.711	1.490
Dizzy/light headed	60/24.5	185/75.5	0.142	0.706	1.064	0.770	1.472
Bloating	88/35.9	157/64.1	0.079	0.778	0.966	0.762	1.226
Weight gain	98/40.0	147/60.0	0.024	0.876	0.983	0.789	1.224
Water retention	66/26.9	179/73.1	0.009	0.924	1.015	0.752	1.369
Tiredness/Fatigue	157/64.1	88/35.9	0.272	0.602	0.965	0.846	1.102
Lack of coordination	54/22.0	191/78.0	0.200	0.655	1.083	0.764	1.536
Sleep disruption	119/48.6	126/51.4	1.161	0.281	0.907	0.760	1.083
Hot flushes/sweating	59/24.1	186/75.9	0.727	0.394	1.158	0.826	1.623
Breast pain/tenderness	89/36.3	156/63.7	0.667	0.414	1.109	0.864	1.424
Cravings	105/42.9	140/57.1	0.007	0.934	1.009	0.818	1.244
Reduced appetite	40/16.3	205/83.7	0.866	0.352	1.230	0.794	1.905
Flooding/leaking	64/26.1	181/73.9	1.547	0.214	1.230	0.886	1.707
Blood clots	58/23.7	187/76.3	0.081	0.776	1.049	0.754	1.459
Mood changes	114/58.8	101/41.2	1.539	0.215	1.107	0.942	1.301
Feeling irritable	160/65.3	85/34.7	0.434	0.510	1.047	0.913	1.200
Feeling emotional	175/71.4	70/28.6	2.216	0.137	1.098	0.970	1.243

Feeling worried	160/65.3	85/34.7	0.434	0.510	1.047	0.913	1.200
Feeling distracted	148/60.4	97/39.6	0.689	0.406	1.067	0.916	1.242
Reduced concentration	155/63.3	90/36.7	1.385	0.239	1.091	0.943	1.264
Lack of motivation	167/68.2	78/31.8	0.515	0.473	1.048	0.922	1.191
Lack of focus	155/63.3	90/36.7	4.583	**0.032**	1.182	1.013	1.379
Reduced sex drive	75/30.6	170/69.4	0.297	0.586	1.081	0.817	1.431
Increased sex drive	65/26.5	180/73.5	0.108	0.743	0.952	0.702	1.279

B

Symptom	Change in Symptom (n/%)	No change in Symptom (n/%)	Chi Squared	P value	Odds ratio	95% Confidence Intervals	
						Lower	Upper
Lower Back pain	7/14.0	43/86.0	5.022	**0.025**	0.409	0.181	0.926
Headaches	13/26.0	37/74.0	0.330	0.565	0.823	0.425	1.596
Nausea/Vomiting	8/16.0	42/84.0	0.371	0.543	0.760	0.314	1.841
Diarrhoea	10/20	40/80	0.173	0.677	0.844	0.381	1.871
Constipation	6/12	44/88.0	0.026	0.871	0.912	0.301	2.765
Dizzy/light headed	16/32.0	34/68.0	0.734	0.392	1.351	0.671	2.719
Bloating	22/44.0	28/56.0	0.032	0.859	1.045	0.642	1.700
Weight gain	15/30.0	35/70.0	0.176	0.674	0.877	0.476	1.616
Water retention	15/30.0	35/70.0	0.011	0.915	1.036	0.539	1.993
Tiredness/Fatigue	31/62.0	19/38.0	0.405	0.524	1.122	0.783	1.607
Lack of coordination	10/20.0	40/80.0	0.035	0.853	1.086	0.455	2.589
Sleep disruption	27/54.0	23/46.0	0.743	0.389	0.855	0.601	1.217
Hot flushes/sweating	11/22.0	39/78.0	3.167	0.075	0.557	0.290	1.072
Breast pain/tenderness	17/34.0	33/66.0	0.605	0.437	0.808	0.472	1.381
Cravings	24/48.0	26/52.0	0.092	0.761	1.073	0.680	1.693

Reduced appetite	14/28.0	36/72.0	0.031	0.861	1.064	0.532	2.128
Flooding/leaking	12/24.0	38/76.0	0.397	0.529	1.303	0.567	2.992
Blood clots	11/22.0	39/78.0	2.010	0.156	2.090	0.721	6.055
Mood changes	26/52.0	24/48.0	1.096	0.295	0.823	0.574	1.181
Feeling irritable	28/58.0	22/44.0	0.182	0.670	0.925	0.648	1.320
Feeling emotional	30/60.0	20/40.0	0.309	0.578	0.912	0.661	1.259
Feeling worried	28/56.0	22/44.0	0.645	0.422	1.182	0.780	1.791
Feeling distracted	26/52.0	24/48.0	0.003	0.953	0.988	0.661	1.477
Reduced concentration	29/58.0	21/42.0	0.557	0.455	1.160	0.781	1.723
Lack of motivation	35/70.0	15/30.0	6.895	**0.009**	1.663	1.098	2.517
Lack of focus	26/52.0	24/48.0	0.185	0.667	1.098	0.715	1.684
Reduced sex drive	13/26.0	37/74.0	0.095	0.758	0.898	0.454	1.779
Increased sex drive	15/30.0	35/70.0	0.895	0.344	1.425	0.675	3.008
